# Current Status and Future Perspectives of Artificial Intelligence in Colonoscopy

**DOI:** 10.3390/jcm11102923

**Published:** 2022-05-22

**Authors:** Yu Kamitani, Kouichi Nonaka, Hajime Isomoto

**Affiliations:** 1 Department of Digestive Endoscopy, Tokyo Women’s Medical University Hospital, 8-1 Kawada-cho, Shinjuku-ku, Tokyo 162-8666, Japan; yukamitani@aol.com; 2 Division of Gastroenterology and Nephrology, Department of Multidisciplinary Internal Medicine, Faculty of Medicine, Tottori University, 36-1 Nishicho, Yonago 683-8504, Japan; isomoto@tottori-u.ac.jp

**Keywords:** artificial intelligence, computer-aided detection/diagnosis, post colonoscopy colorectal cancer, adenoma detection rate

## Abstract

The early endoscopic identification, resection, and treatment of precancerous adenoma and early-stage cancer has been shown to reduce not only the prevalence of colorectal cancer but also its mortality rate. Recent advances in endoscopic devices and imaging technology have dramatically improved our ability to detect colorectal lesions and predict their pathological diagnosis. In addition to this, rapid advances in artificial intelligence (AI) technology mean that AI-related research and development is now progressing in the diagnostic imaging field, particularly colonoscopy, and AIs (i.e., devices that mimic cognitive abilities, such as learning and problem-solving) already approved as medical devices are now being introduced into everyday clinical practice. Today, there is an increasing expectation that sophisticated AIs will be able to provide high-level diagnostic performance irrespective of the level of skill of the endoscopist. In this paper, we review colonoscopy-related AI research and the AIs that have already been approved and discuss the future prospects of this technology.

## 1. Introduction

The prevalence of colorectal cancer is increasing in Asia, including Japan, and worldwide [1,2]. It generally develops according to the adenoma-carcinoma sequence [3], and the early colonoscopy detection, resection, and treatment of colorectal adenoma have been shown to reduce not only the prevalence of colorectal cancer but also its mortality rate [4,5,6,7,8,9,10]. Accordingly, in Japan, it is recommended that individuals aged 40 years or over who test positive for fecal occult blood in health checkups undergo further investigation by total colonoscopy (TCS), and in the United States (US), screening by TCS for all individuals aged 50 years or over who have not previously undergone endoscopic examination is recommended as part of a national project [4]. However, post-colonoscopy colorectal cancer (PCCRC) that develops after TSC is becoming a problem. Cases of PCCRC resulting from a lesion having been overlooked or incompletely resected have been reported [11], and laterally spreading tumors (LSTs) in the right hemicolon and rapidly-growing de novo cancers are said to be particularly easy to overlook. One study has found that an adenoma detection rate (ADR) of <20% significantly increases the risk of PCCRC compared with an ADR of ≥20% [12], and the importance of the ADR as a quality indicator from the viewpoint of picking up lesions has been noted [13]. This situation highlights the significance of aiming for TCS with a low miss rate, and devices such as extra-wide-angle endoscopes and tip hoods are now being used in the attempt to detect lesions in difficult-to-see blind spots. It has also been suggested that image-enhanced endoscopy, such as high-definition endoscopy and chromoendoscopy, may also be useful for the identification of superficial or depressed lesions that are difficult to see even when they are located within the field of view. 

Today, there are increasing expectations for the use of computer-aided diagnosis/detection (CAD) systems that utilize AI. CAD is broadly classified into two categories depending on its purpose: computer-aided detection (CADe) is used to pick up the location of candidate lesions based on the analysis results, and computer-aided detection (CADx) is used to present information on qualitative diagnosis. If sophisticated CAD systems can be developed, in theory, they could provide high-level diagnostic performance irrespective of the level of skill of the endoscopist, and the development of such CAD is thus impatiently awaited. In this review, we place the CAD of colorectal neoplastic lesions under the spotlight, with an overview of prospective studies of CADe and a discussion of trends in research on CADx. We also focus on the current status of commercially available CAD systems that are currently in use in clinical settings and discuss the future prospects for this technology.

## 2. CADe (Computer-Aided Detection)

Computer-aided detection is the use of AI as diagnostic assistance for picking up lesions. As mentioned above, a high ADR is an accepted quality indicator. In one study, a 1% increase in ADR resulted in a 3% decrease in PCCRC and a 5% decrease in fatal PCCRC [13]. Because preventing lesions from being overlooked has been prioritized, it is CADe research that is particularly advanced in AI research in the colorectal field. 

Methods of automatically detecting polyps using a range of different imaging feature quantities (such as edge detection, texture analysis, and energy mapping) had been under investigation since the turn of the millennium, but none of these had reliable detection rates of ≥90%, and limitations on computational capacity meant that none were successful in providing a real-time diagnosis. These methods had the further disadvantage that they only responded to lesion morphology. However, this situation was transformed by the advent of deep learning (DL) in the second decade of the century. 

By adding temporal elements to DL, Misawa et al. developed a convolutional neural network capable of real-time polyp detection with 90.0% sensitivity and 63.3% specificity [14]. Urban et al. also used a large number of static endoscopic images as training images and succeeded in detecting polyps with very high rates of 93.0% sensitivity and 93.0% specificity [15]. 

Subsequently, AI research has proceeded by constructing DL algorithms using retrospectively assembled endoscopic test images, and the performance of these AI systems in clinical settings is currently being analyzed in six prospective randomized clinical trials (Table 1). Five of these six trials have the ADR as the primary endpoint [16,17,18,19,20]. In the other one, the primary endpoint is the adenoma miss rate (AMR) [21]. The most important finding is that in all the trials focusing on a comparison of the ADR, a significant increase in this parameter is evident. To summarize the data from these trials, the ADR increased by 6–15.2% depending on the investigator’s skill and enrollment criteria. In one multicenter joint study using AI, a rate of 40.4% in the control group rose to 54.8% in the CADe group [16]. This suggests that the risk of PCCRC is decreased by assuring a certain ADR [12], and given that the ADR is inversely correlated with mortality [13], the widespread adoption of CAD technology in clinical settings would be highly advantageous. 

According to a more detailed analysis of the randomized clinical trials taking the ADR as the primary endpoint, the detection rate of small adenomas measuring ≤5 mm increased significantly in all these trials [22]. In just one of those trials, the detection rate of adenomas measuring 6–9 mm also rose [16]. Despite the increase in the adenoma detection rate, however, in all the studies there was no significant difference in the scope withdrawal time (excluding polyp resection time) depending on whether CADe support was used or not, a result that is encouraging for its use in actual clinical settings where time is limited. These benefits suggest that the tendency in recent years for the ADR to increase [23] will probably continue and that an increasing number of institutions can be expected to introduce CADe in the future.

## 3. CADx (Computer-Aided Diagnosis)

Computer-aided diagnosis is the use of computer programs for the qualitative diagnosis of lesions and the evaluation of disease activity. Unlike CADe, in which observations are made under normal white light, in CADx not only white-light endoscopy [24,25] but also a wide range of other modalities, including magnifying narrow-band imaging (NBI) [26,27,28,29,30,31], linked-color imaging (LCI) [32], blue-light imaging (BLI) [33,34,35], magnifying chromoendoscopy [36], ultra-high-magnification endoscopy [37,38,39,40,41], confocal laser endoscopy [42,43], autofluorescence spectroscopy [44,45], and autofluorescence imaging (AFI) [46,47,48]. Of these, magnifying NBI has been best studied. The fact that its magnified observations improve diagnostic capability and that time-consuming procedures such as pigment spraying are not required may be an advantage that makes it easy to use in everyday clinical practice. 

Triggered by the start of CADx research on the evaluation of pit patterns by chromoendoscopy, which began after the turn of the millennium [36,49], several research groups have published reports targeting magnified NBI images since 2010 [27]. Some of those studies have come close to achieving practical implementation of real-time diagnosis [26,36]. The diagnostic algorithms used are mainly the methods of using machine learning (such as a support vector machine, neural network, or k-NN classifier) to conduct learning and classification by using a large number of characteristics derived from image filters and texture analysis. In a prospective clinical trial conducted by Kominami et al. [26], the authors validated real-time CADx for 118 lesions in 41 patients, achieving 93.3% sensitivity, and 93.3% specificity. Although that study was small, it is the only prospective study in this research area. 

Since then, DL has garnered attention and become predominant in this field of research. Conventional methods of machine learning require high-level technical skills and knowledge of information engineering in the process of numerically converting endoscopic characteristics, and this constitutes a high hurdle for its development. With DL, however, the process of numerical conversion of these characteristics is simplified, greatly lowering the hurdle for its development; as a result, it has come to be frequently used. Chen et al. [31] and Byrne et al. [30] both succeeded in developing CADx systems using DL that distinguish between tumors and other lesions with better than 90% sensitivity while using the comparatively small number of 3000 images and 300 videos, respectively, as training images based on NBI imaging. This accuracy could be further improved by using a larger number of training cases, and its validation in future prospective studies is awaited. 

The majority of polyps discovered in screening colonoscopy are small lesions measuring ≤5 mm, and it is rare for these small polyps to exhibit neoplastic growth and malignant transformation [50,51]. Polypectomy and pathological investigations themselves may therefore entail a disproportionate burden in terms of cost and effort [52], and a CADx system with high diagnostic performance might enable the choice between “resect and discard” and “diagnose and leave” to be made from the viewpoints of time and cost as well as complications. 

The target of most studies in this field is to distinguish between neoplastic and non-neoplastic lesions, but Tamai et al. [53] used 121 colorectal lesions to develop a CADx system to distinguish those with deep submucosal invasion (T1b cancer), reporting that it distinguished them with 83.9% sensitivity and 82.6% specificity. Colorectal T1b cancer is not easy to distinguish, and as the diagnostic accuracy of clinicians is known to be under 80% [54], the practical implementation of this CAD system could be highly beneficial in clinical practice.

## 4. Commercial CAD Systems

Several CAD systems are already commercially available (Table 2). The main studies of CADe and CADx for commercialization are also summarized (Table 3 and Table 4).

**Figure 1:**
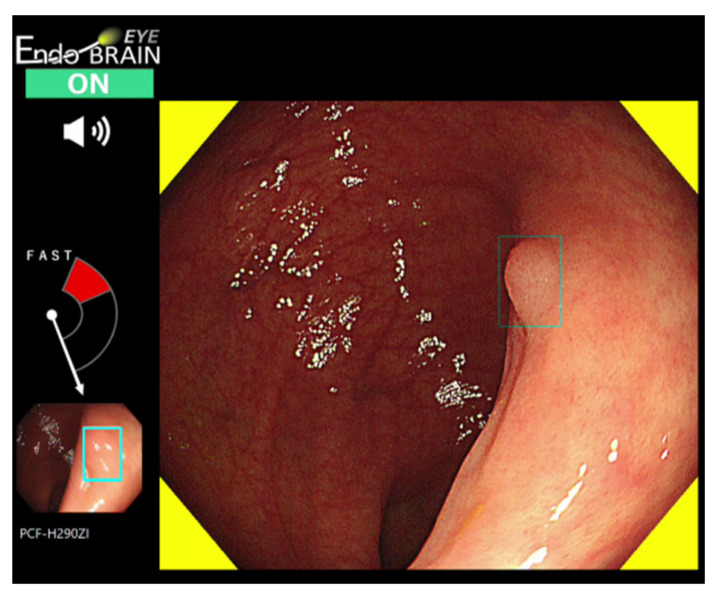
EndoBRAIN-EYE output screen showing the detection of a colorectal polyp. Color and sound alert the user to the detection of a polyp. The location of the polyp is indicated by a rectangle displayed on the screen.

The first of these CADx systems is the EndoBRAIN, jointly developed by Kudo and Mori et al., with Cybernet Systems Co., Japan. It is based on the combination of ultra-high-magnification endoscopy offering up to 520× magnification and a machine-learning algorithm. A study of 205 test lesions that compared the diagnostic accuracy with which it differentiated between neoplastic and non-neoplastic lesions with that of a specialist clinician found that the difference between the EndoBRAIN and the specialist was not significant (EndoBRAIN 89% vs. specialist clinician 91%, *p* = 0.106) [39]. It has undergone performance evaluation tests with a view to approval under the Japanese Pharmaceuticals and Medical Devices (PMD) Act, and a multicenter joint study involving five academic centers has been conducted to establish the EndoBRAIN’s diagnostic accuracy. A comparative study of its accuracy was conducted using an image-interpretation format for 100 colorectal polyps, with images prepared from endoscopic investigations using white-light observations, ultra-high-magnification observations with NBI, and ultra-high-magnification observations with methylene blue staining. The primary endpoints were the sensitivity and accuracy of differentiation between neoplastic and non-neoplastic lesions, and whether the EndoBRAIN provided significantly better results than did non-specialist clinicians. This trial was conducted for 6 months from October 2017, and the results showed that the EndoBRAIN was significantly better than non-specialist clinicians (EndoBRAIN: 97% sensitivity, 98% accuracy vs. non-specialist clinicians: 71% sensitivity, 69% accuracy) [55]. The results of this performance evaluation test were summarized, and it was approved for use as a medical device in Japan in December 2018. Mori et al. have since conducted a large-scale prospective study of the EndoBRAIN. In that trial, it was used for real-time CADx with ultra-high-magnification endoscopy in 791 patients, and the pathological diagnosis was predicted for a total of 466 diminutive colorectal polyps. The results demonstrated that it is capable of distinguishing neoplastic lesions with 92.7% sensitivity and 89.8% specificity, indicating that this CADx system is useful in actual clinical practice. In the field of CADe, the EndoBRAIN-EYE has been developed, using an Olympus general-purpose colonoscope for the automatic detection of colorectal lesions and providing real-time support during the investigation (Figure 1). In a frame-based analysis of 152,560 endoscopy images (49,799 positive images containing polyps and 102,761 negative images not containing polyps), the AI achieved good results with 90.5% sensitivity and 93.7% specificity. After completing clinical performance trials, it was approved for use as a medical device in Japan in January 2020 and was launched on the market in May of that year [56]. Taking this one step beyond the distinction between neoplastic and non-neoplastic lesions, a new EndoBRAIN-Plus has also been developed that is capable of diagnosing invasive cancer, and this underwent an evaluation of its diagnosis of invasive cancer using 200 endoscopic images extracted from a database. This CAD system uses ultra-high (approximately 400×) magnification endocytoscopy (EC-CAD). The results showed that of these 200 lesions, 188 (94.0%) were evaluable by the EC-CAD system. The sensitivity, specificity, accuracy, positive predictive value (PPV), and negative predictive value (NPV) were 89.4%, 98.9%, 94.1%, 98.8%, and 90.1%, respectively, indicating that the EC-CAD system was capable of highly reliable diagnosis [41]. The EndoBRAIN-UC, which analyzes ultra-high-magnification endoscopic images of ulcerative colitis and evaluates inflammatory activity, has also been approved [57], and AI may thus be of use for inflammatory bowel disorders as well as neoplastic lesions. The EndoBRAIN-Plus and EndoBRAIN-UC went on the market in Japan in February 2021.

The first CADe system to be commercialized was Medtronic’s GI Genius. In July 2019, it was awarded the CE mark required for its export sales in the European Union (EU), and in 2021, it was approved by the US Food and Drug Administration (FDA). The CADe system was developed by Cosmo Pharmaceuticals, and a multicenter joint randomized clinical trial was conducted by Hassan and Repici et al. [16]. In its use in the real-time endoscopy of 685 patients divided into a CADe group and a control group examined by experts without the use of CADe, the ADR was significantly higher in the CADe group (54.8%) than in the control group (40.4%).

The DISCOVERY system (Pentax Medical) is another CADe system that was also awarded the CE mark in January 2020 and is considered to be capable of detecting flat lesions including typical adenomas and serrated polyps, but no clinical trial has been conducted at this point.

The ENDO-AID system (Olympus) is a CADe system that can automatically detect polyps, cancer, and other candidate lesions and display them in real-time when used in combination with the EVIS X1 endoscopy system introduced by the company in April 2020. Since its approval in the EU in November 2020, it has been on sale in Europe and some parts of Asia. This CAD has also not been subject to any clinical trial at this point.

**Figure 2:**
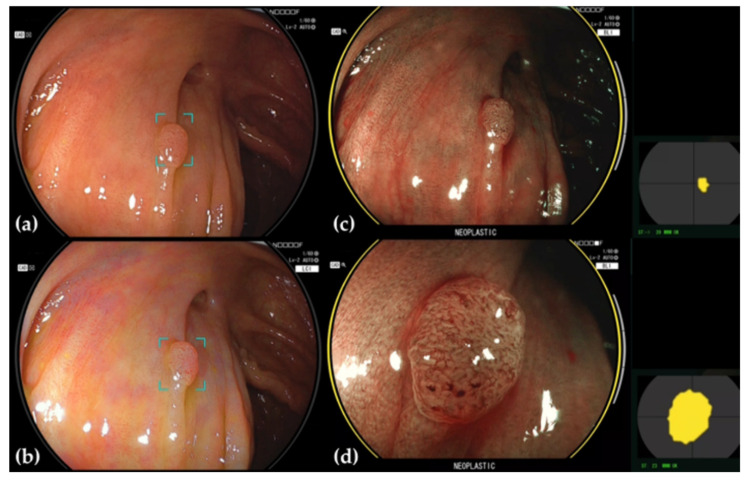
Adenoma detected and diagnosed by CADEYE. (**a**) The margins of the area around a suspected polyp detected under WLE are delineated. (**b**) This area is similarly delineated under LCI. (**c**) If the lesion is determined to be neoplastic under BLI, the endoscopic image is ringed in yellow, and the word “NEOPLASTIC” is displayed beneath. The location where the determination is being conducted is also shown to the right of the endoscopy image. (**d**) Magnified BLI image.

**Figure 3:**
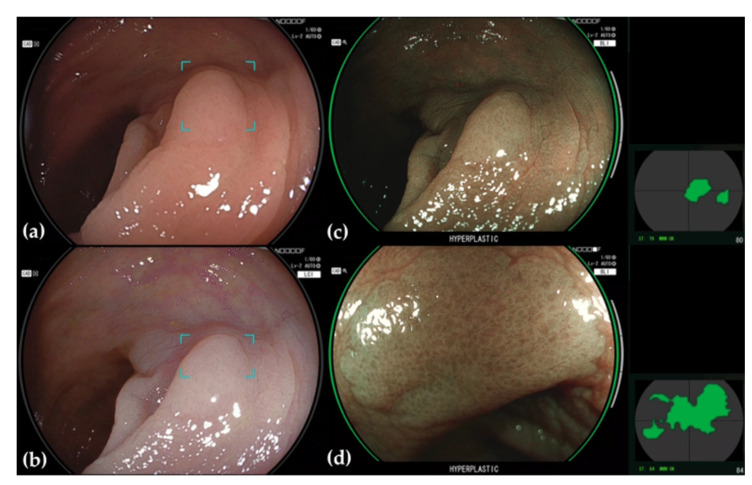
Hyperplasic polyp detected and diagnosed by CADEYE. (**a**) The margins of the area around a suspected polyp detected under WLE are delineated. (**b**) This area is similarly delineated under LCI. (**c**) If the lesion is determined to be hyperplastic under BLI, the endoscopic image is ringed in green, and the word “HYPERPLASTIC” is displayed beneath. The location where the determination is being conducted is also shown to the right of the endoscopy image. (**d**) Magnified BLI image.

CAD EYE (Fujifilm) is the first CAD system to include both CADe and CADx systems as part of the same platform, incorporating a CADe system that helps to detect polyps by using LCI, a function that intensifies and displays slight differences in color in the red zone, in addition to white-light imaging (WLI), and a CADx system that uses BLI to distinguish polyps by intensifying and displaying minute vessels and structures in the mucosal surface of organs by varying the light emission ratios of multiple lights with different wavelengths (Figure 2 and Figure 3). Its effectiveness was evaluated in a retrospective performance trial of colorectal polyps using endoscopic images obtained from four centers in Europe and three in Japan as validation images. The primary endpoint for CADe in this evaluation was sensitivity, and that for CADx was accuracy [34,35]. For the CADe system, the detection sensitivities of WLI and LCI were 94.5% and 96.0%, respectively, and the 95% confidence intervals did not go below 90% for either. CADx can be used in either WLI or BLI mode, and in the performance evaluation test, its accuracy was 93.2% with WLI and 94.9% with BLI, indicating the utility of this system. Taking into account the differential diagnostic capacity when magnified endoscopy is used, as these results were obtained when the system was in operation without magnification, they were satisfactory results for the performance evaluation stage. The system was awarded the CE mark in the EU in February 2020, and it was approved under the PMD Act in Japan in September 2020. Since then, a number of CAD reports have investigated LCI and BLI, which are characteristic of Fujifilm. One study that focused on LCI investigated its sensitivity (true-positive rate per lesion) and false-positive frame rate using video data including 240 polyps photographed in LCI mode. The results showed that it had 100% sensitivity without a single miss, and the false-positive frame rate was extremely low at 0.001%. Of these 240 polyps, 34 lesions were sessile serrated lesions, but the detection rate on LCI was still 100% [60]. There are two different types of BLI, blue-laser imaging (BLI-LASER) and blue-light imaging (BLI-LED), and the sensitivity, specificity, and accuracy of non-magnified BLI-LASER/LED when CADx was used were 91.7%, 86.8%, and 88.8%, respectively. For magnified BLI-LASER/LED, a comparison between the CADx and trainees showed that accuracy was significantly higher for the CADx (79.0% vs. 87.8%, *p* = 0.04), and a comparison between the CADx and experts found no significant difference between them (92.0% vs. 87.8%, *p* = 0.17), suggesting that CADx provides a high level of performance. That study also compared the diagnostic accuracy of the CADx with LED and LASER imaging and found that although the accuracy was somewhat higher with LED, the difference was not significant (95.7% vs. 81.4%, *p* = 0.07). This was an extremely interesting study, which included both experts and trainees and evaluated performance using both LED and LASER endoscopy [33].

The EndoScreener system (Shanghai Wision AI Co., China) is a CADe system jointly developed by Wang et al. In six randomized controlled trials (RCTs), including three open RCTs [17,20], one double-blind RCT [18], and two tandem colonoscopy RCTs [21,59], the improved adenoma detection rate was demonstrated in over 5000 patients. In the latest multi-center tandem colonoscopy trial conducted at four academic medical centers in the US, the AMR was significantly lower in the EndoScreener-first group compared with the high-definition white light (HDWL) colonoscopy-first group (20.12% vs. 31.25%, *p* = 0.0247), and the SSL miss rate was also significantly lower in the EndoScreener-first group compared with the HDWL colonoscopy-first group (7.14% vs. 42.11%, *p* = 0.0482). The adenoma per colonoscopy (APC) rate was also significantly higher in the EndoScreener-first group (1.19 vs. 0.90, *p* = 0.0323) [59]. In view of the results of these RCTs, in November 2021 it was awarded the first CE mark (Class II) under the new medical device regulations (MDR 2017/745) and approved by the FDA in the US.

WISE VISION is a CADe system jointly developed by the Japanese National Cancer Center and NEC. The AI in this software was trained on 250,000 endoscopic images (static or video) of more than 10,000 early-stage colon cancer or precancerous lesions with findings annotated by endoscopists and is characterized by focused deep learning on superficial and depressed tumors that are particularly difficult to identify. In a study conducted by Yamada et al., this AI system had 97.3% sensitivity and 99% specificity, and subgroup analysis showed that it had 98.1% sensitivity for elevated lesions and 92.9% sensitivity for superficial or depressed lesions [58]. Similar results were obtained in performance evaluation tests, and it received approval in Japan under the PMD Act in November 2020 and was awarded the CE Mark in the EU in December of that year.

## 5. Limitations and Future Perspective

As discussed above, CAD may have a very major role to play in the detection and diagnosis of colorectal tumors, but whether its use will contribute to improving survival rates for colorectal cancer has yet to be determined. Most of the actual data used in CAD systems has been put together from colonoscopy examinations carried out in hospitals and consists of a mixture of both outpatient and inpatient data. Of the prospective RCTs of CADe so far conducted, one was carried out in a single European country and the other five in one specific Asian country. Differences in race, lifestyle factors, underlying conditions, purposes of investigation, and other factors mean that use of CADe may not necessarily be indicated for all situations. In the results of the European RCT, the baseline ADR differed markedly from those of the other five Asian RCTs [16,17,18,19,20,21]. The clinical trials conducted in Asia were all single-center trials, and a simple comparison between studies with restricted subject populations with multicenter studies carried out in other regions may not be possible. In addition, although these trials found that the ADR was improved by CAD use, there was no significant increase in the detection rate of advanced adenoma (size ≥ 10 mm, possessing a villiform component on histopathological examination, and high-grade dysplasia) [61]. High-grade dysplasia accounts for less than 1% of diminutive polyps, defined as a size of <6 mm [62], and it is currently unclear whether the natural history of this category of polyps follows the same course as that of the development of PCCRC from adenomas measuring ≥ 10 mm. From these results, the key point is currently the improved rate of detection of diminutive polyps, but a calm discussion of whether this in fact leads to clinically significant results, such as improved patient prognosis, may be required. Future studies should perhaps focus on whether the use of CAD incorporating AI can demonstrate improved survival as a result of decreased PCCRC in worldwide multicenter studies.

In terms of CADx, some systems incorporate functions to distinguish whether detected lesions are neoplastic or non-neoplastic. The use of these diagnostic support functions not only reduces the burden on expert endoscopists but has also been reported to enable non-experts to achieve sensitivity and accuracy that are not inferior to those of experts by their concomitant use of CADx [34]. However, they do have several limitations. The first is that SSLs, which are regarded as precancerous lesions, are diagnosed in an equivalent way to hyperplastic polyps, which are non-neoplastic, and for this reason, data on the differences between SSLs and hyperplastic polyps have been lacking from CADx trials conducted so far. This is because SSLs and hyperplastic polyps have similar surface structures as seen in endoscopies. CADx systems, therefore, require further appropriate training to be able to distinguish between them. Secondly, some lesions cannot be recognized because of the size, shape, or position of the tumor. Improvements in the ability of both CADe and CADx to recognize lesions such as submucosal tumors, the color of which resembles that of normal mucosa. Thirdly, data on the differentiation between T1 cancer and adenoma, and to go one step further, on the differentiation between T1a cancer and T1b cancer, are still limited. This is an important issue because the qualitative diagnosis of these tumors is directly linked to treatment strategy in terms of the choice between endoscopic mucosal resection (EMR) and endoscopic submucosal dissection (ESD), and the choice between endoscopic or surgical treatment. As mentioned above, Takeda et al. [41] and Tamai et al. [53] both presented highly accurate data on invasive cancer, but as data sufficient to distinguish between T1a cancer and T1b Cancer are lacking, the assumption is that these systems can only ever be used as an auxiliary diagnosis. Further studies on the diagnosis of invasion depth are awaited.

Beyond the diagnosis of cancer invasion depth, the introduction of AIs to predict metastasis and recurrence is now under investigation. Kudo and Ichimasa et al. are developing an AI capable of cancer metastasis prediction. They produced an AI trained by machine learning with a support vector machine, using not only the endoscopic findings and histopathological diagnoses of 3134 T1 cancer patients but also clinical data such as blood test results. In external validation tests, the AI model identified patients with lymph node metastasis with an area under the receiver operating characteristics curve (AUC) of 0.83, whereas the guidelines used as a comparative control identified them with an AUC of 0.73 (*p* < 0.001), indicating that this AI model is capable of predicting lymph node metastasis of T1 cancer with a high degree of accuracy [63]. Takamatsu et al. are developing an AI to predict the metastatic potential of T1 cancer from image analysis of pathological specimens obtained by endoscopic or surgical resection. They created a training dataset using machine learning based on lymph node metastasis results for 277 of 397 T1 cancer patients and predicted the occurrence of lymph node metastasis in the remaining 120 patients from their test data. They reported that it predicted lymph node metastasis with equivalent or greater accuracy compared with the conventional method [64], and the further development of this AI is anticipated.

## 6. Conclusions

In this paper, we reviewed reported studies of the use of AI in colonoscopy and the current status of and future prospects for AIs that are currently commercially available and approved for use as medical devices. The speed with which the development of endoscopic AI has proceeded recently is eye-opening, and several products have already cleared regulatory hurdles and are available for use in everyday clinical practice. There have been numerous reports of their accuracy bearing comparison with that of specialist endoscopists, and whether endoscopy AIs will eventually be able to out-perform specialists is now the subject of debate. However, there are limitations to the mechanisms of AI themselves, and it may, unfortunately, be difficult to create a versatile AI that is both universal and provides better accuracy than that of doctors. Endoscopists will thus not be replaced by AI but will need to live with them, understanding the strengths and weaknesses of individual Ais and using them wisely.

## Figures and Tables

**Table 1:** Prospective randomized trial analyses in CADe systems.

	Wang et al. (2019) [20]	Wang et al. (2020) [21]	Wang et al. (2020) [18]	Lui et al. (2020) [17]	Su et al. (2020) [19]	Repici et al. (2020) [16]
**Study design**	single center non-blinding	single center non-blinding	single center double-blind	single center non-blinding	single center non-blinding	multicenter non-blinding
**Material**						
**Image modality**	HD ^(1)^ video /WLE ^(2)^	HD video /WLE	HD video /WLE	HD video /WLE	HD video /WLE	HD video /WLE
**Vendor**	Olympus	Fujifilm	Fujifilm	Olympus	Pentax Medical	Fujifilm /Olympus
**Endoscopist**	various levels	experienced	experienced	n/a ^(3)^	experienced	experienced
**Population**						
**Patients, n**	1058	369	962	790	623	685
**Age, mean**	50	47	49	49	51	61
**Screening** **CS ^(4)^**	7.9%	30.6%	16.5%	23%	34.7%	46.3%
**Results** **in CADe**						
**Primary endpoint**	ADR ^(5)^	AMR ^(6)^	ADR	ADR	ADR	ADR
**Withdrawal time**	6.9 min	6.5 min	6.5 min	6.7 min	7.0 min	7.1 min
**ADR**	29% IRR ^(7)^ 1.61	42.4% IRR 1.33	34% IRR 1.36	29.0% IRR 1.55	28.9% IRR 2.06	54.8% IRR 1.35
**APC ^(8)^**	0.53 IRR 1.72	0.78 IRR 1.2	0.58 IRR 1.53	0.48 IRR 1.64	0.37 IRR 2.06	1.07 IRR 1.46
**PPC ^(9)^**	0.95 IRR 1.89	1.55 IRR 1.17	1.04 IRR 1.61	1.07 IRR 2.09	0.58 IRR 1.89	1.88 IRR 1.54
**SDR ^(10)^**	3.41%	0.35%	3.6%	0.8%	n/a	7.0%
**Increase** **of CRC ^(11)^**	no	no	no	no	no	no

^(1)^ High-definition, ^(2)^ White light endoscopy, ^(3)^ not available, ^(4)^ Colonoscopy, ^(5)^ Adenoma detection rate, ^(6)^ Adenoma miss rate, ^(7)^ Incidence rate ratio, ^(8)^ Adenomas per colonoscopy, ^(9)^ Polyps per colonoscopy, ^(10)^ Sessile serrated lesion detection rate, ^(11)^ Colorectal cancer.

**Table 2:** Overview of commercial CAD systems.

Product Name	Company	Integration	Study Data	CAD Mode	Regulatory (Year, Region)
**EndoBRAIN**	Cybernet Systems Co. (Tokyo, Japan)	CF-H290ECI, Olympus Co.	Mori Y et al. [39] Kudo S et al. [55]	CADx	2018, Japan
**EndoBRAIN-EYE**	Cybernet Systems Co.	Olympus colonoscopes	Misawa M et al. [56]	CADe	2020, Japan
**EndoBRAIN-PLUS**	Cybernet Systems Co.	CF-H290ECI, Olympus Co.	Takeda K et al. [41]	CADx	2020, Japan
**EndoBRAIN-** **UC**	Cybernet Systems Co.	CF-H290ECI, Olympus Co.	Maeda Y et al. [57]	CADx	2020, Japan
**GI Genius**	Medtronic Co. (Dublin, Ireland)	Multi vendors possible	Repici A et al. [16]	CADe	2019, EU/USA
**DISCOVERY**	Pentax Medical Co. (Tokyo, Japan)	Pentax colonoscopes	n/a ^(1)^	CADe	2020, EU
**ENDO-AID**	Olympus Co. (Tokyo, Japan)	Olympus colonoscopes	n/a	CADe	2020, EU
**CAD EYE**	Fujifilm (Tokyo, Japan)	Fujifilm colonoscopes	Weigt J et al. [34]	CADe, CADx	2020, EU/Japan
**EndoScreener**	Shanghai Wision AI Co. (Shanghai, China)	Multi vendors possible	Wang P et al. [21]	CADe	2021, EU/USA
**WISE VISION**	NEC Co. (Tokyo, Japan)	Multi vendors possible	Yamada M et al. [58]	CADe	2020, Japan/EU

^(1)^ not available.

**Table 3:** Study data of commercial CADe systems.

Product Name	Author	Study Design	Modality	Results
**EndoBRAIN-EYE**	Misawa M et al. [56]	retrospective	WLI ^(1)^	sensitivity/specificity 90.5%/93.7%
**GI Genius-**	Repici A et al. [16]	prospective	WLI	ADR ^(2)^ (CADe vs. Control) 54.5% vs. 40.4%
**CAD EYE**	Weigt J et al. [34]	retrospective	WLI/LCI ^(3)^	sensitivity (WLI/LCI) 94.5%/96.0%
**EndoScreener**	Wang P et al. [21] Brown J.R.G et al. [59]	prospective tandem study	WLI	ADR (CADe first vs. routine first) 42.4% vs. 35.7% AMR ^(4)^ (CADe first vs. routine first) 20.12% vs. 31.25% SSL ^(5)^ miss rate (CADe first vs. routine first) 7.14% vs. 42.11% APC ^(6)^ (CADe first vs. routine first) 1.19 vs. 0.90
**WISE VISION**	Yamada M et al. [58]	retrospective	WLI	sensitivity/specificity 97.3%/99% Subgroup analysis for sensitivity elevated lesions 98.1% superficial or depressed lesions 92.9%

^(1)^ White-light imaging, ^(^^2)^ Adenoma detection rate, ^(3)^ Linked-color imaging, ^(4)^ Adenoma miss rate, ^(5)^ Sessile serrated lesion detection rate, ^(6)^ Adenomas per colonoscopy.

**Table 4:** Study data of commercial CADx systems.

Product Name	Author	Study Design	Modality	Results
**EndoBRAIN**	Mori Y et al. [39] Kudo et al. [55]	retrospective retrospective	EC ^(1)^ EC	accuracy (CADx vs. specialist clinician) 89% vs. 91% sensitvity (CADx vs. non-specialist) 97% vs. 71% accuracy (CADx vs. non-specialist) 98% vs. 69%
**EndoBRAIN-** **PLUS**	Takeda K et al. [41]	retrospective	EC	sensitivity 89.4%, specifity 98.9%, accuracy 94.1%, PPV ^(2)^ 98.8%, NPV ^(3)^ 90.1%
**CAD EYE**	Weigt J et al. [34]	retrospective	WLI ^(4)^, BLI ^(5)^	accuracy 93.2% (WLI), 94.9% (BLI)

^(1)^ Endocytoscopy, ^(2)^ Positive predictive value, ^(3)^ Negative predictive value, ^(4)^ White-light imaging, ^(5)^ Blue-light imaging.

## Data Availability

Not applicable.
